# Predictors of Persistent Fever Among Patients With Suspected Infective Endocarditis: Think Outside the box

**DOI:** 10.1093/cid/ciae588

**Published:** 2024-11-27

**Authors:** Elisavet Stavropoulou, Pierre Monney, Georgios Tzimas, Nicoleta Ianculescu, Piergiorgio Tozzi, Matthias Kirsch, Benoit Guery, Matthaios Papadimitriou-Olivgeris

**Affiliations:** Infectious Diseases Service, Lausanne University Hospital and University of Lausanne, Lausanne, Switzerland; Department of Cardiology, Lausanne University Hospital and University of Lausanne, Lausanne, Switzerland; Department of Cardiology, Lausanne University Hospital and University of Lausanne, Lausanne, Switzerland; Department of Cardiology, Lausanne University Hospital and University of Lausanne, Lausanne, Switzerland; Department of Cardiac Surgery, Lausanne University Hospital and University of Lausanne, Lausanne, Switzerland; Department of Cardiac Surgery, Lausanne University Hospital and University of Lausanne, Lausanne, Switzerland; Infectious Diseases Service, Lausanne University Hospital and University of Lausanne, Lausanne, Switzerland; Infectious Diseases Service, Lausanne University Hospital and University of Lausanne, Lausanne, Switzerland; Infectious Diseases Service, Cantonal Hospital of Sion and Institut Central des Hôpitaux, Sion, Switzerland

**Keywords:** infective endocarditis, persistent bacteremia, persistent fever, spondylodiscitis, source control

## Abstract

**Background:**

Fever is common in infective endocarditis (IE), yet little is known about fever duration in such patients. We aim to identify predictors of persistent fever in patients with suspected IE.

**Methods:**

This study was conducted at the Lausanne University Hospital, Switzerland, from January 2014 to June 2023. All patients with suspected IE being febrile upon presentation were included. Fever (>38°C) was considered persistent if it continued for at least 96 hours from antimicrobial treatment initiation. A case was classified as IE by the Endocarditis Team.

**Results:**

Among 1399 episodes with suspected IE, persistent fever was observed in 260 (19%) episodes. IE was diagnosed in 536 (41%) episodes, of which 82 (15%) had persistent fever. Among episodes with suspected IE, persistent bacteremia/candidemia for 96 hours (*P* < .001), spondylodiscitis (*P* = .039), intrabdominal infection (*P* = .001) were associated with persistent fever. Conversely, bacteremia by streptococci (*P* = .049), or enterococci (*P* = .001), source control performed withing 96 hours (*P* = .015) and appropriate antimicrobial treatment within 48 hours (*P* = .018) were associated with early defervescence. No association between persistent fever and infective endocarditis was found (*P* = .207). Among 536 IE episodes, persistent bacteremia/candidemia for 96 hours (*P* < .001), and native bone and joint infection (*P* = .020) were associated with persistent fever. Conversely, bacteremia by streptococci or enterococci (*P* = .001; adjusted odds ratio [aOR] 0.25, 95% confidence interval [CI] .11–.58) were associated with early defervescence.

**Conclusions:**

In episodes with suspected IE, persistent fever was associated with spondylodiscitis, inappropriate antimicrobial treatment and absence of source control interventions. Among IE patients, persistent fever was associated with native bone and joint infections.

## Teaser

Persistent fever was observed in 19% of episodes with suspected infective endocarditis. No association between persistent fever and infective endocarditis was found. However, spondylodiscitis, inappropriate antimicrobial treatment, and absence of early source control were associated with persistent fever.

Fever is the most prevalent clinical sign among the many clinical manifestations of infective endocarditis (IE) [1, 2]. Due to its high prevalence, fever was included as a minor criterion in the original Duke criteria for diagnosing IE and remains part of subsequent updates [3–5].

In patients with suspected IE, fever prevalence is similar in both those who are ultimately diagnosed with IE and those who are not [6]. However, in certain contexts, such as *Staphylococcus aureus* bacteremia, persistent fever may indicate a more complicated disease and should raise the suspicion of IE [7, 8]. Despite this, a previous study from our institution found that persistent fever rates among patients with suspected IE did not significantly differ between those who were later confirmed to have IE and those who were not [9]. This indicates that in suspected cases of IE, persistent fever may be influenced by factors unrelated to IE.

The course of fever after the initiation of antimicrobial treatment among patients with diagnosed IE has not been adequately addressed. Retrospective studies conducted prior to 2000 found that persistent fever among IE patients, despite antimicrobial therapy, was associated factors to extensive cardiac involvement [10–12], and embolic events [12, 13]. Less common causes include extracardiac sources of infection, polymicrobial endocarditis, drug-induced fever, or nosocomial infections [10, 14]. Patients with persistent fever were significantly more likely to have life-threatening complications, such as paravalvular invasion, and large vegetations, leading to poorer clinical outcomes [10, 11, 13, 15–17].

Despite advancements in the diagnosis and management of IE, contemporary data on the duration and persistence of fever in patients with suspected or confirmed IE remain limited. This study aims to assess whether persistent fever is associated with IE in patients with suspected IE and to identify predictors of persistent fever in patients who are diagnosed with IE.

## METHODS

### Study Design

This single-center study was conducted at Lausanne University Hospital, Switzerland, from January 2014 to June 2023 (2014–17: retrospective cohort of IE patients; 2018 onward: prospective cohort of patients with suspected IE). The Ethics Committee of the Canton of Vaud has approved the study (CER-VD 2017-02137, CER-VD 2021-02516).

### Patients

All adult patients (≥18 years old) with suspected IE (patients who had blood cultures drawn and an echocardiography performed specifically for the research of IE) and fever upon presentation were included. Exclusion criteria were absence of documentation of temperature measurements or death within 96 hours from antimicrobial treatment initiation without 24 hours of defervescence. Additional exclusion criteria were for the retrospective cohort refusal to use their data and for the prospective cohort the absence of written consent.

Demographic, clinical, microbiological, radiological, and surgical data were retrieved through patient's electronic health charts. Per internal guidelines, all suspected IE cases underwent infectious diseases (ID) consultation with a thorough physical examination. Follow-up blood cultures were taken every 24–48 hours until clearance. IE diagnosis was made by the Endocarditis Team based on clinical, laboratory, microbiological, imaging, surgical, and histopathological results. The determination of other foci of infection was based on the assessment by the ID consultant, taking into account clinical, radiological, microbiological, and operative findings. In case of fever, patients received paracetamol as needed.

### Definitions

Fever was defined according to Duke criteria as a temperature superior to 38°C (>100.4°F). Persistent fever was defined as continuous fever for at least 96 hours from antimicrobial treatment initiation. Persistent bacteremia/candidemia was defined as continued positive blood cultures for at least 96 hours from antimicrobial treatment initiation. Cases were classified as definite, possible, and rejected IE based on the 2023 International Society of Cardiovascular Infectious Diseases (ISCVID) version of the Duke criteria [5]. Sepsis and septic shock were defined as per the Sepsis-3 International Consensus [18]. Antimicrobial treatment was considered appropriate if the patient received within 48 hours at least 1 active antimicrobial agent for which the isolated pathogen was considered susceptible. Source control was considered warranted in the following situations: (1) removal of venous catheter in patients with catheter-related bacteremia or bacteremia of unknown origin with the presence of a venous catheter; (2) imaging-guided or surgical drainage of infected collections; (3) joint fluid drainage (arthrotomy, arthroscopy, needle aspiration); (4) cardiac surgery in endocarditis patients when indicated for heart failure [19]; (5) correction of urinary-tract obstruction.

### Statistical Analyses

SPSS version 26.0 (SPSS, Chicago, Illinois, USA) software was used for data analyses. Patients were categorized according to fever duration as “no persistent fever” and “persistent fever.” Group differences were investigated using the Mann–Whitney *U* test for continuous variables and the χ^2^ [2] or Fisher exact test for categorical variables. Univariable analyses were performed with persistent fever as dependent variable. Covariates were tested for multi-collinearity through variance inflation factor assessment: those not collinear and clinically relevant were used in multivariate logistic regression analyses. The strength of any association was calculated as adjusted odds ratios (aORs) and 95% confidence intervals (CIs). All tests were 2-tailed, and *P* < .05 was considered statistically significant.

## RESULTS

### Cohort Composition

**Figure 1.**
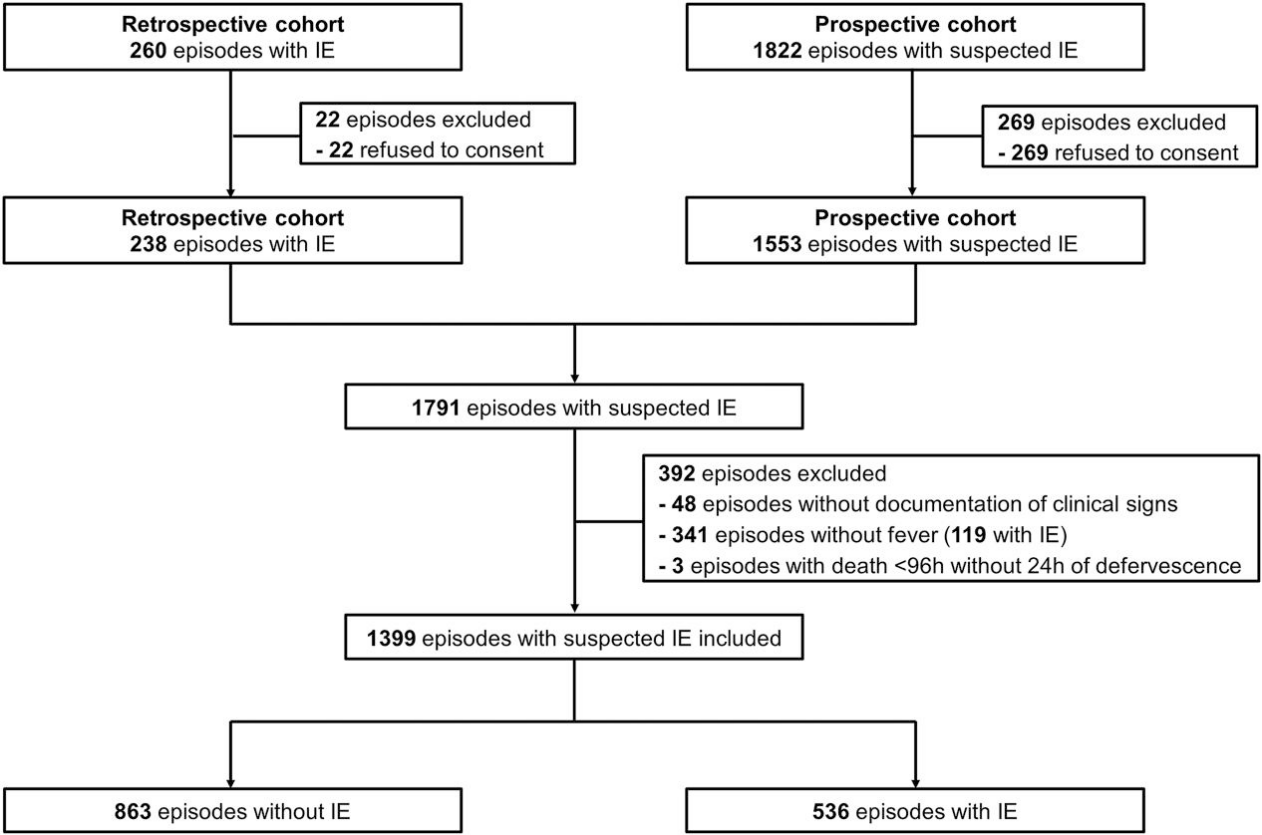
Flowchart of patients. Abbreviation: IE, infective endocarditis.

Among 2082 episodes with suspected IE, 1399 episodes were included (Figure 1); among different reasons of exclusion, 341 episodes of suspected IE were excluded due to absence of fever upon presentation (Figure 1). From 1399 included episodes, 536 (38%) had IE, and for 863 (62%) IE diagnosis was excluded. In total, 105 (8%) episodes had a non-infectious diagnosis (malignancy-related fever, auto-immune disease, fever of unknown origin, non-infective endocarditis, etc). Transthoracic, transesophageal echocardiography (TTE, TEE), ^18^F-Fluorodeoxyglucose positron emission tomography/computed tomography (CT), and cardiac CT were performed in 1339 (96%), 694 (50%), 293 (21%), and 54 (4%) episodes, respectively. The first echocardiography was performed within a median of 3 days (interquartile range [IQR]: 1–5 days) from presentation. Among patients with *S. aureus* bacteremia first echocardiography was performed within a median of 3 days (interquartile range; IQR: 1–4 days).

### Persistent Fever Among Episodes With Suspected IE

Among 1399 episodes with suspected IE, persistent fever was observed in 260 (19%) episodes. Table 1 depicts predictors of persistent fever among patients with suspected IE. The median duration of fever was 1 day (IQR: 1–3 days) from antimicrobial treatment initiation. Spondylodiscitis (8% vs 5%; *P* = .050), intraabdominal infection (9% vs 3%; *P* = .001), persistent bacteremia/candidemia for 96 hours (15% *vs* 5%; *P* < .001) and fungemia (5% *vs* 3%; *P* = .029) were more prevalent among episodes with persistent fever, compared to those without. In contrast, IE was more prevalent among episodes without persistent fever compared to those with (40% vs 32%; *P* = .013). Bacteremia by either streptococci (19% *vs* 12%; *P* = .005) or enterococci (12% vs 5%; *P* =.001) were less prevalent among episodes with persistent fever, compared to those with early defervescence. Of the 1278 (out of 1399; 91%) episodes requiring antimicrobial treatment, a smaller proportion of cases with persistent fever received within 48 hours an appropriate therapy compared to those without persistent fever (91% vs 95%; *P* = .004). Source control procedures were necessary in 583 episodes (42%); these interventions were less frequently performed within 96 hours in episodes with persistent fever compared to those without (24% vs 59%; *P* = .015).

**Table 1.** Predictors of Persistent Fever Among Patients With Suspected Infective Endocarditis

	No Persistent Fever (n = 1139)	Persistent Fever (n = 260)	*P*
Demographics			
Male sex	819 (72)	175 (67)	.150
Age (y)	68 (54–78)	65 (55–75)	.022
Age >60 y	751 (66)	158 (61)	.130
Co-morbidities			
Malignancy (solid organ or hematologic)	222 ()20	77 (30)	.001
Obesity	289 (25)	69 (27)	.694
Immunosuppression	367 (32)	98 (38)	.094
Manifestations			
Sepsis	431 (38)	105 (40)	.480
Septic shock	125 (11)	28 (11)	1.000
Cardiac predisposing factors			
IV drug use	69 (6)	19 (7)	.479
Prior endocarditis	67 (6)	10 (4)	.229
Congenital disease	82 (7)	11 (4)	.097
Prosthetic valve	211 (19)	34 (13)	.038
Cardiac implantable electronic device	152 (13)	18 (7)	.003
Non-cardiac prosthetic material			
Vascular catheter (central or peripheral)	440 (39)	110 (42)	.291
Prosthetic joint	178 (16)	45 (17)	.511
Vascular graft	93 (8)	23 (9)	.709
Final diagnosis			
Non-infectious diagnosis	74 (7)	31 (12)	
Infectious diagnosis	1065 (94)	229 (88)	.004
Bacteremia/candidemia of unknown origin	96 (8)	18 (7)	.530
Catheter-related	124 (11)	28 (11)	1.000
Infective endocarditis	454 (40)	82 (32)	.013
Skin and soft tissue infection	112 (10)	22 (9)	.560
Bone and joint infection	226 (20)	62 (24)	.149
Native bone and joint infection	123 (11)	39 (15)	.067
Septic arthritis	75 (7)	22 (9)	.280
Spondylodiscitis	56 (5)	21 (8)	.050
Low-respiratory tract infection	57 (5)	17 (7)	.356
Urinary tract infection	49 (4)	13 (5)	.617
Intrabdominal infection	39 (3)	22 (9)	.001
Other type of infection	128 (11)	25 (10)	.509
Microbiological data			
Bacteremia/candidemia	930 (82)	184 (71)	<.001
*S. aureus*	409 (36)	99 (38)	.521
Coagulase negative staphylococci	71 (6)	9 (4)	.102
Streptococci	215 (19)	30 (12)	.005
Enterococci	137 (12)	14 (5)	.001
Other Gram-positive	34 (3)	6 (2)	.682
HACEK	13 (1)	3 (1)	1.000
Other Gram-negative	93 (8)	22 (9)	.900
Fungi	30 (3)	14 (5)	.029
Polymicrobial bacteremia/candidemia	80 (7)	11 (4)	.124
Persistent bacteremia/candidemia for 96h	56 (5)	99 (15)	<.001
Source control warranted	469 (41)	114 (44)	.471
Source control performed within 96h^a^	275 (59)	52 (24)	.015
Antimicrobial treatment warranted	1053 (93)	225 (88)	.003
Appropriate antimicrobial treatment within 48h^b^	1005 (95)	204 (91)	.004

Data are depicted as number (percentage) or median (interquartile range).

Abbreviation: HACEK, *Haemophilus* spp, *Aggregatibacter* spp, *Cardiobacterium hominis*, *Eikenella corrodens*, *Kingella kingae*; IV, intravenous; *S. aureus*, *Staphylococcus aureus*.

^a^Among 583 episodes.

^b^Among 1278 episodes.

Multivariable logistic regression among the 1399 episodes with suspected IE (Table 2) identified malignancy, either solid organ or hematological (*P* = .012; aOR 1.89, 95% CI: 1.15–3.12), persistent bacteremia/candidemia for 96 hours (*P* < .001; aOR 2.55, 95% CI: 1.46–3.49), spondylodiscitis (*P* = .039; aOR 1.79, 95% CI: 1.03–3.12), intrabdominal infection (*P* = .001; aOR 2.86, 95% CI: 1.55–5.27) as factors associated with persistent fever. Conversely, bacteremia by streptococci (*P* = .049; aOR 064, 95% CI .41–.99), or enterococci (*P* = .001; aOR 0.37, 95% CI: .20–.69), source control performed within 96 hours (*P* = .015; aOR 0.58, 95% CI: .38–.90) and appropriate antimicrobial treatment within 48 hours (*P* = .018; aOR 0.60, 95% CI: .39–.92) were associated with early defervescence.

**Table 2.** Bivariate and Multivariable Analyses of Predictors of Persistent Fever Among Patients With Suspected Infective Endocarditis

	Bivariate Analysis		Multivariable Analysis	
	*P*	OR (95% CI)	*P*	aOR (95% CI)
Malignancy (solid organ or hematologic)	.001	1.74 (1.28–2.36)	.012	1.89 (1.15–3.12)
Immunosuppression	.094	1.27 (.96–1.68)	.309	.79 (.49–1.25)
Bacteremia by streptococci	.005	.56 (.37–.84)	.049	.64 (.41–.99)
Bacteremia by enterococci	.001	.42 (.24–.73)	.001	.37 (.20–.69)
Fungemia	.029	2.10 (1.10–4.03)	.242	1.52 (.75–3.07)
Persistent bacteremia/candidemia for 96h^a^	<.001	2.49 (1.69–3.69)	<.001	2.55 (1.46–3.49)
Spondylodiscitis	.050	1.70 (1.01–2.86)	.039	1.79 (1.03–3.12)
Infective endocarditis	.013	.70 (.52–.93)	.207	.80 (.57–1.13)
Intrabdominal infection	.001	2.61 (1.52–4.48)	.001	2.86 (1.55–5.27)
Source control				
Warranted; not performed within 96h	reference	reference	reference	reference
Warranted; performed within 96h	.015	.59 (.39–.89)	.015	.58 (.38–.90)
Not warranted	.030	.68 (.49–.96)	.122	.75 (.52–1.09)
Antimicrobial treatment				
Warranted; not appropriate within 48h^a^	reference	reference	reference	reference
Warranted; appropriate within 48h	.008	.46 (.27–.79)	.018	.60 (.39–.92)
Not warranted	1.000	.99 (.51–1.94)	.917	1.04 (.51–2.12)

Abbreviations: aOR, adjusted odds ratios; CI, confidence interval; OR, odds ratio.

### Persistent Fever Among Episodes With IE

Among 655 IE episodes with documented temperature upon presentation, 536 episodes were included, although 119 (18%) episodes were excluded due to absence of fever at presentation. Of the 536 included IE episodes, 82 (15%) exhibited persistent fever. The median duration of fever among IE episodes was 1 day (IQR: 1–3 days) from antimicrobial treatment initiation (Supplementary Table 1). Native bone and joint infections (27% vs 14%; *P* = .008), embolic events (51% *vs* 37%; *P* = .020), persistent bacteremia/candidemia for 96 hours (39% *vs* 11%; *P* < .001) and bacteremia by *S. aureus* (67% vs 38%; *P* < .001) were more prevalent among episodes with persistent fever, compared to those without (Table 3). Bacteremia by either streptococci or enterococci (45% vs 12%; *P* < .001) was less prevalent among episodes with persistent fever, compared to those with early defervescence. Cardiac lesions (vegetations ≥10 mm, intracardiac abscess) or type of valve (native or prosthetic) were not associated with persistent fever. The median duration of fever among episodes with either vegetation ≥10 mm, or intracardiac abscess was 1 day (IQR: 1–3 days) (Supplementary Table 1); even by excluding the episodes that had cardiac surgery within 4 days from antimicrobial treatment initiation the duration of fever for patients with vegetation ≥10 mm was 1 day (IQR: 1–3 days), and for intracardiac abscess 1 day (IQR: 1–4 days).

**Table 3.** Characteristics of Patients With and With no Persistent Fever Among Patients With Infective Endocarditis

	No Persistent Fever (n = 454)	Persistent Fever (n = 82)	*P*
Demographics			
Male sex	348 (77)	60 (73)	.485
Age (y)	68 (54–77)	66 (51–76)	.257
Age >60 y	301 (66)	50 (61)	.378
Comorbidities			
Congestive heart failure	53 (12)	6 (7)	.337
Chronic obstructive pulmonary disease	48 (11)	14 (17)	.094
Cirrhosis	27 (6)	6 (7)	.619
Diabetes mellitus	108 (24)	23 (28)	.405
Chronic kidney disease (moderate or severe)	362 (20)	15 (18)	.765
Malignancy (solid organ or hematologic)	54 (12)	9 (11)	1.000
Obesity	113 (25)	21 (26)	.890
Immunosuppression	91 (20)	19 (23)	.553
Cardiac predisposing factors	291 (64)	45 (55)	.136
Microbiological data			
*S. aureus*	173 (38)	55 (67)	<.001
Coagulase negative staphylococci	30 (7)	4 (5)	.805
Streptococci	134 (30)	10 (12)	.001
Enterococci	71 (16)	0 (0)	<.001
Other Gram-positive	13 (3)	1 (1)	.706
HACEK	12 (3)	3 (4)	.713
Other Gram-negative	13 (3)	3 (4)	.722
Intracellular bacteria	4 (.9)	0 (0)	1.000
Fungi	5 (1)	4 (5)	.035
Persistent bacteremia/candidemia for 96h	50 (11)	32 (39)	<.001
Manifestations			
Sepsis	204 (45)	49 (60)	.016
Septic shock	77 (17)	19 (23)	.210
Embolic events	170 (37)	42 (51)	.020
Cerebral	92 (20)	19 (23)	.555
Non-cerebral	124 (27)	32 (39)	.035
Immunologic phenomena	33 (7)	12 (15)	.048
Native bone and joint infection	65 (14)	22 (27)	.008
Septic arthritis	42 (9)	14 (17)	.047
Spondylodiscitis	27 (6)	11 (13)	.031
Type of diagnosis^a^			
Definite IE	300 (66)	59 (72)	.311
Possible IE	154 (34)	23 (28)	
Site of infection			
Aortic valve	237 (52)	40 (49)	.631
Mitral valve	182 (40)	36 (44)	.543
Tricuspid valve	34 (8)	11 (13)	.084
Pulmonary valve	9 (2)	2 (2)	.679
Multivalvular	47 (10)	11 (13)	.439
CIED-IE	61 (13)	6 (7)	.147
Other site of infection	5 (1)	2 (2)	.292
Type of valve			
Native	299 (66)	55 (67)	.899
Prosthetic	121 (27)	24 (29)	.685
Intracardiac lesions			
Vegetation	304 (67)	55 (67)	1.000
Vegetation ≥10mm	165 (36)	33 (40)	.535
Abscess	83 (18)	18 (22)	.444
Other lesions^b^	79 (17)	14 (17)	1.000
Source control warranted	172 (38)	40 (49)	.067
Source control performed within 96h^c^	76 (17)	15 (18)	.482
Appropriate antimicrobial treatment within 48h	429 (95)	75 (92)	.309

Data are depicted as number (percentage) or median (interquartile range).

Abbreviations: CIED, cardiac implantable electronic devices; HACEK, *Haemophilus* spp, *Aggregatibacter* spp, *Cardiobacterium hominis*, *Eikenella corrodens*, *Kingella kingae*; IE, infective endocarditis; ISCVID, International Society of Cardiovascular Infectious Diseases; *S. aureus*, *Staphylococcus aureus*.

^a^According to the 2023 ISCVID version of the Duke criteria.

^b^Perforation, dehiscence of prosthetic valve, fistula, pseudoaneurysm, aneurysm.

^C^Among 212 episodes.

In multivariable logistic regression (Table 4), persistent bacteremia/candidemia for 96 hours (*P* < .001; aOR 2.71, 95% CI: 1.54–4.77), and native bone and joint infections (*P* = .020; aOR 2.07, 95% CI: 1.12–3.83) were associated with persistent fever among IE episodes. Conversely, bacteremia by streptococci or enterococci (*P* = .001; aOR 0.25, 95% CI: .11–.58) were associated with early defervescence.

**Table 4.** Bivariate and Multivariable Analyses of Predictors of Persistent Fever Among Patients With Infective Endocarditis

	Bivariate Analysis		Multivariable Analysis	
	*P*	OR (95% CI)	*P*	aOR (95% CI)
Bacteremia by *S. aureus*	<.001	3.31 (2.01–5.44)	.890	.96 (.50–1.84)
Bacteremia by streptococci or enterococci	<.001	.17 (.09–.34)	.001	.25 (.11–.58)
Persistent bacteremia/candidemia for 96h	<.001	5.17 (3.04–8.81)	<.001	2.71 (1.54–4.77)
Sepsis	.016	1.81 (.12–2.93)	.343	1.30 (.76–2.21)
Embolic events	.020	1.75 (1.09–2.82)	.120	1.54 (.89–2.67)
Native bone and joint infection	.008	2.19 (1.26–3.82)	.020	2.07 (1.12–3.83)
Immunological phenomena	.048	2.19 (1.08–4.44)	.331	1.52 (.66–3.52)
Tricuspid valve infective endocarditis	.084	1.91 (.93–3.95)	.996	.99 (.45–2.23)

Abbreviations: aOR, adjusted odds ratios; CI, confidence interval; OR, odds ratio; *S. aureus*, *Staphylococcus aureus*.

## DISCUSSION

In our cohort of episodes with suspected IE, persistent fever was observed in 19% of episodes, with no association between IE and persistent fever.

As previously reported, fever was a common clinical sign of IE [1, 2]. In our study, 81% of episodes with suspected IE and 82% of IE episodes presented with fever, consistent with findings from 2 large IE studies; 78% of IE cases had fever in the EURO-ENDO registry [1] and 96% in the International Collaboration on Endocarditis-Prospective Cohort Study [2]. Although fever is a minor Duke criterion for diagnosing IE [3–5], a study of 3127 episodes with suspected IE found the prevalence of fever to be similar among patients with confirmed IE (78%) and those whose suspicion of IE was ultimately ruled out (79%), indicating that fever alone lacks the discriminatory power to distinguish IE from non-IE cases [6].

As expected, among episodes with suspected IE in general and those with IE, persistent fever was associated with persistent bacteremia/candidemia, as both are hallmarks of uncontrolled infection. Successful management of infection relies on two core components: appropriate antimicrobial treatment and timely source control. In our study, most episodes with suspected IE requiring antimicrobial therapy received appropriate treatment within 48 hours, which was associated with early resolution of fever. Similarly, among episodes with suspected IE that necessitated source control, performing these interventions promptly was linked to early defervescence. As previously demonstrated in studies on *S. aureus* bacteremia, timely source control not only accelerated the clearance of bacteremia but also reduced mortality [20, 21]. This emphasizes the clinical importance of early fever resolution as an indicator of effective infection management. In cases of *S. aureus* bacteremia, fever persistence beyond 72 hours is a marker of complicated infection, along with persistent bacteremia and lack of source control. The presence of any of these criteria necessitates extending antibiotic therapy from 2 weeks to 4–6 weeks [7].

In suspected IE cases, certain infections, such as spondylodiscitis, were also linked to persistent fever in IE patients. Spondylodiscitis often coincides with paravertebral or epidural abscesses, which are typically small and do not cause progressive neurological deficits, spinal instability, or significant deformity, and therefore do not always require surgical intervention [22]. However, even small abscesses can continue to act as a source of bacterial spread due to reduced antimicrobial penetration. Persistent bacteremia despite appropriate antimicrobial therapy indicates the need for source control in patients with spondylodiscitis [22]. These findings align with the established association between spondylodiscitis and IE [23], as reflected in risk prediction models for *S. aureus* bacteremia (VIRSTA and LAUSTAPHEN scores) [24, 25].

In cases with diagnosed IE, few studies have examined the course of persistent fever, with none conducted in the past two decades. Historically, Douglas et al reported that 36% of 83 IE cases had persistent fever (>7 days), although they used a lower temperature threshold (37.1°C or >98.8°F) [10]. Other retrospective studies reported that fever persisted for more than 7 days in 28% of 123 and 36% of 193 IE cases [12, 13]. However, in the present study, only 15% of IE episodes presented with persistent fever (≥4 days). It is worth noting that in most of these earlier studies, IE was diagnosed using the von Reyn criteria, and the definition of persistent fever varied in terms of temperature cut-off and duration [10, 12, 13]. Despite these methodological differences, the duration of fever in all prior studies was significantly longer than in the present study, where the median duration was only 1 day. Interestingly, in contrast to older studies, large vegetations (≥10 mm), intracardiac abscesses, and embolic events were not associated with prolonged fever in our cohort, even after excluding patients that had early valve surgery [10, 11, 13]. For example, the median duration of fever was 1 day for patients with vegetations (IQR: 1–3 days), intracardiac abscesses (IQR: 1–3 days), and those with embolic events (IQR: 1–3 days). This is in stark contrast to the study by Lederman et al, which reported fever lasting 10 days in patients with vegetations and 9 days in those with embolic events [13]. Thus, early defervescence or early clearance of blood cultures, especially in non-staphylococcal IE cases [26], should not rule out the presence of intracardiac complications. A TEE should be performed on all patients with IE, or high suspicion of IE, even if there is rapid clinical improvement following the initiation of antimicrobial treatment [19]. The discrepancies in the duration of fever in the present study compared to those conducted previously could be explained by an advancements in diagnostic tools (microbiological and radiological) over the past three decades, allowing for earlier identification of patients with less advanced intracardiac invasion. In our study, patients with *S. aureus* bacteremia had the first echocardiography within 3 days of the first positive blood culture, compared to 5.7 days in a study conducted more than 20 years ago [27]. Earlier identification of IE patients has led to improved outcomes and reduced mortality by enabling earlier management of such patients [28].

Moreover, IE episodes caused by *S. aureus* were more likely to exhibit persistent fever, consistent with earlier findings [10, 12, 13], although this association was not confirmed in multivariable logistic regression. This suggests that other factors, such as persistent bacteremia or native bone and joint infections like spondylodiscitis, may mediate the relationship [24, 25]. Conversely, bacteremia caused by streptococci and enterococci was associated with a lower likelihood of persistent fever in both suspected IE and confirmed IE cases. Previous studies also demonstrated that fever duration was shorter in IE caused by enterococci and streptococci compared to *S. aureus* IE or culture negative IE [12, 13, 29].

This study has several limitations. First, it is a single-center study; however, to our knowledge, it is the largest study to date that explores persistent fever in patients with suspected IE and the only one conducted in the last 2 decades. Second, because this study was performed in a Swiss university hospital where ID specialists evaluate all suspected IE cases, with access to advanced imaging such as ^18^F-FDG PET/CT and cardiac CT for valvular and paravalvular lesions, as well as comprehensive imaging for embolic events, the findings may have limited generalizability to other healthcare systems. Third, due to the lack of a definitive gold standard for diagnosing IE, the Endocarditis Team's evaluation was used as the reference standard. Fourth, we did not account for medications such as paracetamol, anti-inflammatory drugs, or corticosteroids, which can affect body temperature. Fifth, we did not collect data on subfebrile temperatures (37.5–38°C), which in our study were considered afebrile. Therefore, we cannot exclude the possibility that patients with cardiac complications may have remained subfebrile for a longer duration than those without such complications.

In conclusion, our study challenges the traditional association between persistent fever and IE, because no association between persistent fever and IE among patients with suspected IE was found, with most patients achieving defervescence withing 4 days of initiating antimicrobial treatment. Among episodes with suspected IE, persistent fever was linked to inappropriate antimicrobial treatment, lack of source control interventions, and spondylodiscitis, whereas among IE patients, it was associated with native bone and joint infections. Notably, in these patients, large vegetations (≥10 mm), intracardiac abscess, or embolic events were not associated with persistent fever. These findings underscore the importance of reassessing patients with persistent fever despite appropriate antimicrobial therapy, which may require repeated imaging and consideration of source control procedures. Identifying these predictors can guide necessary investigations and optimize intervention timing, ultimately improving patient outcomes.

## Supplementary Material

ciae588_Supplementary_Data

## Supplementary Data

Supplementary materials are available at *Clinical Infectious Diseases* online. Consisting of data provided by the authors to benefit the reader, the posted materials are not copyedited and are the sole responsibility of the authors, so questions or comments should be addressed to the corresponding author.

## Notes

***Author Contributions***. M. P. O., P. M., and B. G. conceived the idea. E. S., P. M., G. T., N. I., P. T., M. K., B. G., and M. P. O. collected the patients' data. M. P. O. supervised the project. M. P. O. performed the analysis and interpreted the results. E. S. wrote the manuscript. All authors contributed to manuscript revision and read and approved the submitted version.
