# Supplementary material for: Predictors of Persistent Fever Among Patients With Suspected Infective Endocarditis: Think Outside the box
Source: Clin Infect Dis. 2024 Nov 27;80(4):795–803. doi: 10.1093/cid/ciae588 (PMC12043059; doi:10.1093/cid/ciae588)
Supplement: ciae588_Supplementary_Data [file ciae588_supplementary_data.docx]

Supplementary Table 1. Fever duration in various scenarios of infective endocarditis

**Characteristics**	**Duration of fever (days)**
S. aureus	2 (1-4)
Streptococci	1 (1-2)
Enterococci	1 (1-2)
Embolic events	2 (1-3)
Vegetation	1 (1-3)
Vegetation ≥10mm	1 (1-3)
By excluding patients operated within 4 days from antimicrobial treatment	1 (1-4)
Intracardiac abscess	1 (1-3)
By excluding patients operated within 4 days from antimicrobial treatment	1 (1-3)

Data are depicted as median (interquartile range)
